# Bazedoxifene and conjugated estrogen combination maintains metabolic homeostasis and benefits liver health

**DOI:** 10.1371/journal.pone.0189911

**Published:** 2017-12-21

**Authors:** Karen Lee Ann Chen, Yiru Chen Zhao, Kadriye Hieronymi, Brandi Patricia Smith, Zeynep Madak-Erdogan

**Affiliations:** 1 Division of Nutritional Sciences, UIUC, Urbana, Illinois, United States of America; 2 Department of Food Science and Human Nutrition, UIUC, Urbana, Illinois, United States of America; 3 Institute for Genomic Biology, UIUC, Urbana, Illinois, United States of America; 4 National Center for Supercomputing Applications, UIUC, Urbana, Illinois, United States of America; Universite du Quebec a Montreal, CANADA

## Abstract

The bazedoxifene and conjugated estrogens (CE+BZA) combination has been shown to prevent visceral adiposity and weight gain after ovariectomy. However, its impact on the liver transcriptomes associated with prevention of hepatosteatosis is yet to be determined. In the present study, we use liver transcriptomics and plasma metabolomics analysis to characterize the effects of various estrogens on liver. The CE+BZA combination was very effective at preventing ovariectomy-induced weight gain in mice fed a high-fat diet (HFD). In CE+BZA treated animals, liver weight and hepatic lipid deposition were significantly lower than in Vehicle (Veh) treated animals. Additionally, CE+BZA induced unique liver transcriptome and plasma metabolome profiles compared to estradiol, conjugated estrogens alone, and bazedoxifene alone. Blood plasma metabolite analysis identified several metabolites similar to and distinct from other estrogen treatments. Integrated pathway analysis showed that gene networks that were associated with inflammation, reactive oxygen species pathway and lipid metabolism and their relevant metabolites were regulated significantly by CE+BZA treatment. Thus, long-term CE+BZA treatment modulated hepatic metabolic gene networks and their associated metabolites and improves hepatic health without stimulating the uterus.

## Introduction

With increased life expectancy, a significant portion of the US population will quickly be over 60 and a significant majority of this population is going to be women [1]. Estrogen deficiency in postmenopausal women increases incidences of cardiovascular disease and skeletal degeneration, as well as, obesity, type 2 diabetes, and non-alcoholic fatty liver disease (NAFLD) [2–4]. In animal models of estrogen-deficiency, these effects, including insulin resistance and glucose tolerance, were improved by estradiol (E2) treatment [5, 6].

Conjugated estrogens (CE) are a mix of various estrogens and estrogen metabolites **(**S1 Fig**)** that are currently used in clinic either alone or in combination with progestin to manage postmenopausal symptoms. Selective estrogen receptor modulators (SERMs) are compounds that bind to estrogen receptors (ER) and exert agonistic or antagonistic activity based on the tissue background. Bazedoxifene (BZA) is one such SERM that has estrogen agonist activity in bone and reduces osteoporosis while having antagonistic activity in the breast and uterus [7, 8]. In mice, BZA blocks effects of E2 in the mammary gland and inhibits the growth of tamoxifen sensitive and resistant breast tumors [9]. In cell culture models, CE was much less potent in inducing breast cancer cell proliferation, and BZA entirely suppressed this effect [10]. CE+BZA combination has been described as tissue-selective estrogen complexes (TSECs). This combination, marketed as Duavee, was approved by the FDA for the prevention of postmenopausal osteoporosis and the treatment of moderate-to-severe vasomotor symptoms caused by menopause [11–13]. In addition to improving postmenopausal symptoms, CE+BZA prevented weight gain due to estrogen deficiency in ovariectomized mice [14, 15]. CE+BZA treated, ovariectomized animals had a significant decrease in both uterine and mesenteric white adipose tissue, yet the treatment did not cause an increase in uterine weight [15]. In mice, CE+BZA treatment decreased plasma leptin levels, leptin/adiponectin ratio, and levels of thiobarbituric acid reactive substances (TBARS) compared to vehicle treatment, suggesting normalization of plasma adipokines and systemic inflammation [14]. Furthermore, the CE+BZA treatment decreased hepatic fatty acid synthase (FAS) enzymatic activity [14] and liver lipid deposition [15].

To understand the molecular and systemic effects of CE+BZA combination on metabolism in the low estrogen environment, we performed liver transcriptomics and plasma metabolomics analysis in ovariectomized female mice. In this well-established estrogen deficiency model [16–19], CE+BZA combination modulated inflammation-related pathways and decreased the expression of gene networks that control lipid deposition in the liver. In summary, our combined liver gene expression and blood metabolite analysis showed that CE+BZA combination is very effective in mitigating weight gain and lipid deposition in liver, by modulating fatty acid metabolism, reactive oxygen species- and inflammation-associated pathways that are deregulated due to loss of estrogens and high-fat diet.

## Material and methods

### Animal model and treatments

**Fig 1:**
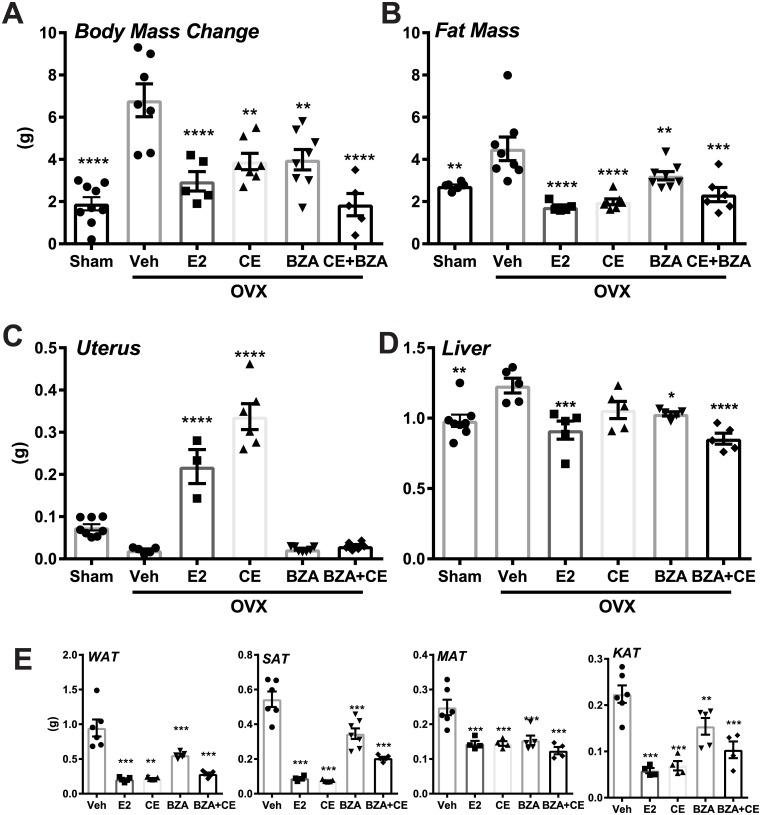
CE+BZA combination decreases ovariectomy-associated weight gain and lipid deposition without stimulating reproductive tissue. (A) Total body weight change was measured at the end of six weeks of various estrogen treatments. (B) Fat mass was measured using EchoMRI at the end of four weeks of various estrogen treatments. (C) Uterus and (D) liver were harvested at the end of six weeks and weighed. (E) WAT, SAT, MAT, and KAT were harvested and weighed. All data were normally distributed. Statistical significance was established at α = 0.05. Pairwise t tests with a Bonferroni correction were used to identify treatments that were significantly different from Veh (*P < 0.05, **P < 0.01, ***P <0.001, ****P<0.0001).

48 female mice, C57BL/6J (RRID:IMSR_JAX:000664), from Jackson Laboratory were housed individually in 12-h light-dark cycle. At 8 weeks of age, mice were switched to a high-fat diet (Harlan TD.88137), which was based on an AIN76a diet but with 45% of calories from fat and 0.2% cholesterol. Mice were given water ad libitum. Based on our previous work on body weight normalization by low affinity estrogens in mice treated with controls or various estrogens, typical treatment differences in the weight of animals on high fat diet following ovariectomy were 5 g; standard deviations were approximately 3 g [10, 18]. Using these predictions and a Type I error of 5% and a Type II error of 10%, we estimated that 8 animals were required for each group in each experiment. Forty mice were ovariectomized (OVX) under isoflurane anaesthesia at ten weeks of age. The rest of the mice were kept as sham operated control group (Sham). Ovariectomized mice were divided randomly into five treatment groups (8 animals per treatment group): (1) vehicle at 43% DMSO, 15% ethanol, and 42% saline; (2) E2 at 5 μg·kg^-^1·day^-1^; (3) CE at 2.5 mg·kg^-^1·day^-1^; (4) BZA at 3mg·kg^-1^·day^-1^; (5) CE+BZA. The dosages of the estrogens were selected based on previously published studies.[14, 15] Conjugated estrogens were centrifuged at 9,000g for 1 minute to remove insoluble cellulose before loading into a 60-day release osmotic minipump as described (Alzet 2006, DURECT Corporation; flow rate 0.15 μl/hr) [14, 15]. This mode of delivery resulted in ~100 pg/ml of E2 and estrone delivery in previous studies [14]. E2 was delivered using 0.72 mg, 60-day release E2 pellets from Innovative Research of America, as we reported previously [20]. E2 and CE supplementation induced the predicted uterotropic responses, whereas animals that were treated with Veh, BZA or CE+BZA were devoid of this response **(**Fig 1C**)**. All experiments involving animals were conducted with protocols approved by the University of Illinois at Urbana-Champaign and by the National Institutes of Health standards for use and care of animals (IACUC Protocol 14193).

### Food intake and body composition

Food intake and body weight were measured weekly for six weeks. MRIs were performed before treatment, and then at four weeks after treatment using an EchoMRI machine, which measures the whole body, lean, and water mass. After six weeks of treatment, mice were euthanized and organs were harvested. Perigonadal white adipose tissue (WAT), mesenteric adipose tissue (MAT), perirenal/kidney adipose tissue (KAT), subcutaneous adipose tissue (SAT), liver, and uterus were weighed.

### Liver histology

Liver tissue sections were embedded in Tissue-Tek O.C.T. compound and frozen. Sections were fixed in 10% NBF then stained with hematoxylin and eosin (H&E) or Oil Red O. Images were quantified using Fiji software. Five liver sections from each group at 2 fields per section were analysed to quantify lipid droplet size and number.

### Real-time RT-qPCR

Liver tissue from each animal (8 animals/treatment group) was homogenized in 1 mL of TRIzol reagent, and total RNA was isolated. RNA was transcribed into cDNA using M-MuLV Reverse Transcriptase (BioLabs). Fast Start Universal SYBR Green reagent (Roche) and an Applied Biosystem Step One Plus qPCR system were used for the RT-qPCR reaction. Results were normalized to Rplp0 as the internal reference gene, and the relative difference in gene expression from the vehicle was calculated using the ΔΔCt method. PCR primer sequences were obtained from PrimerBank (https://pga.mgh.harvard.edu/primerbank/)

### RNA-seq and transcriptional profiling

Three liver RNA samples from each treatment group were selected. RNA was cleaned using an RNAeasy kit (Qiagen). Concentrated RNA was submitted to the DNA Sequencing Group at the Roy J. Carver Biotechnology Center at UIUC. cDNA libraries were prepared with the mRNA TruSeq Kit (Illumina Inc.). Double stranded cDNA was generated from fragmented RNA and adapters were ligated to the ends. Casava 1.8.2. was used to base call and demultiplex samples. FASTQ files were trimmed using FASTQ Trimmer (version 1.0.0). TopHat (version 0.5) was used to map single-end RNA-seq reads to *Mus musculus* reference genome. Gene expression values quantified from BAM files were calculated using StrandNGS (version 3.1) Quantification tool. Partial reads were considered and the option of detecting novel genes and exons was selected. Default parameters for finding novel exons and genes were specified. DESeq normalization algorithm using default values was chosen. Differentially expressed genes were then determined by fold-change and p-value with Benjamini and Hochberg multiple test correction for each gene for each treatment relative to the vehicle control. We considered genes with fold-change >2 and FDR (or q) < 0.05 as significantly and differentially expressed. PCA analysis was performed using StrandNGS.

### Metabolic profiling

500 ul of whole blood was extracted from abdominal aorta at the time of tissue harvest. Ten μl of 0.5 M EDTA was added to blood samples and samples were centrifuged for 10 minutes at room temperature at 1,000 x g. Supernatant was removed and stored at -80°C. Plasma samples from each animal were submitted to the Metabolomics Center in the Roy J Carver Biotechnology Center (CBC) at UIUC. GC/MS whole metabolite profiling was performed to detect and quantify the metabolites by using Gas chromatography-mass spectrometry (GC/MS) analysis. Metabolites were extracted from 50 μl of plasma according to Agilent Inc. application notes. Hentriacontanoic acid was added to each sample as the internal standard prior to derivatization. Metabolite profiles were acquired using an Agilent GC-MS system (Agilent 7890 gas chromatograph, an Agilent 5975 MSD, and an HP 7683B autosampler). The spectra of all chromatogram peaks were evaluated using the AMDIS 2.71 and a custom-built database with 460 unique metabolites. All known artificial peaks were identified and removed prior data mining. To allow comparison between samples, all data were normalized to the internal standard in each chromatogram. Metabolomic data was integrated with gene expression data and analysed by Pathway Analysis modules in Cytoscape using the ClueGo application and Metaboanalyst.

### Statistical analyses

Data from gene expression, metabolomics and animal metabolism studies were analysed using either a one-way analysis of variance (ANOVA) model to compare different ligand effects, a two-way-ANOVA model to compare time-dependent changes. All data was tested for normal distribution using pairwise t tests with a Bonferroni correction. All data was normally distributed unless otherwise noted. Normally distributed data was analysed using pairwise t tests with a Bonferroni correction to identify treatments that were significantly different from each other (*P < 0.05, **P < 0.01, ***P <0.001, ****P <0.0001). For every main effect that was statistically significant at α = 0.05, pairwise t-tests were conducted to determine which ligand treatment levels were significantly different from each other. For these t-tests, the Bonferroni correction was employed to control experiment wise type I error rate at α = 0.05 followed by Bonferroni post hoc test. Data that were not normally distributed were analyzed using Mann Whitney test for nonparametric data (*P < 0.05, **P < 0.01, ***P <0.001, ****P <0.0001). Statistical significance was calculated using GraphPad Prism for Windows (GraphPad Software, La Jolla California USA, www.graphpad.com).

### Availability of data and materials

Gene expression data is available at GEO database under the accession number GSE92968.

## Results

### CE+BZA is very effective in decreasing ovariectomy-associated weight gain and lipid deposition without stimulating reproductive tissue

To characterize the liver specific effects of various estrogens, ovariectomized female mice on HFD were treated with vehicle, CE, BZA or CE+BZA in combination for 6 weeks. Sham operated (Sham) or E2 treatment groups were included as controls. Ovariectomy increased body weight **(**Fig 1A**)**, fat mass **(**Fig 1B**)** and liver weight **(**Fig 1D**)**, while uterus weight decreased **(**Fig 1C**)**. Various estrogen supplementations effectively reduced body weight, fat mass, liver, and various adipose depot weights, including WAT, SAT, MAT and KAT **(**Fig 1E**)**. Uterus weight is only increased by E2 and CE treatments. Total body weight of ovariectomized mice that were fed a high fat diet was significantly decreased in E2, CE only, BZA only and CE+BZA groups compared to the animals in Veh group. These data suggest that CE+BZA treatment prevents low-estrogen and high-fat diet induced weight gain and lipid deposition without stimulating the uterus.

**Fig 2:**
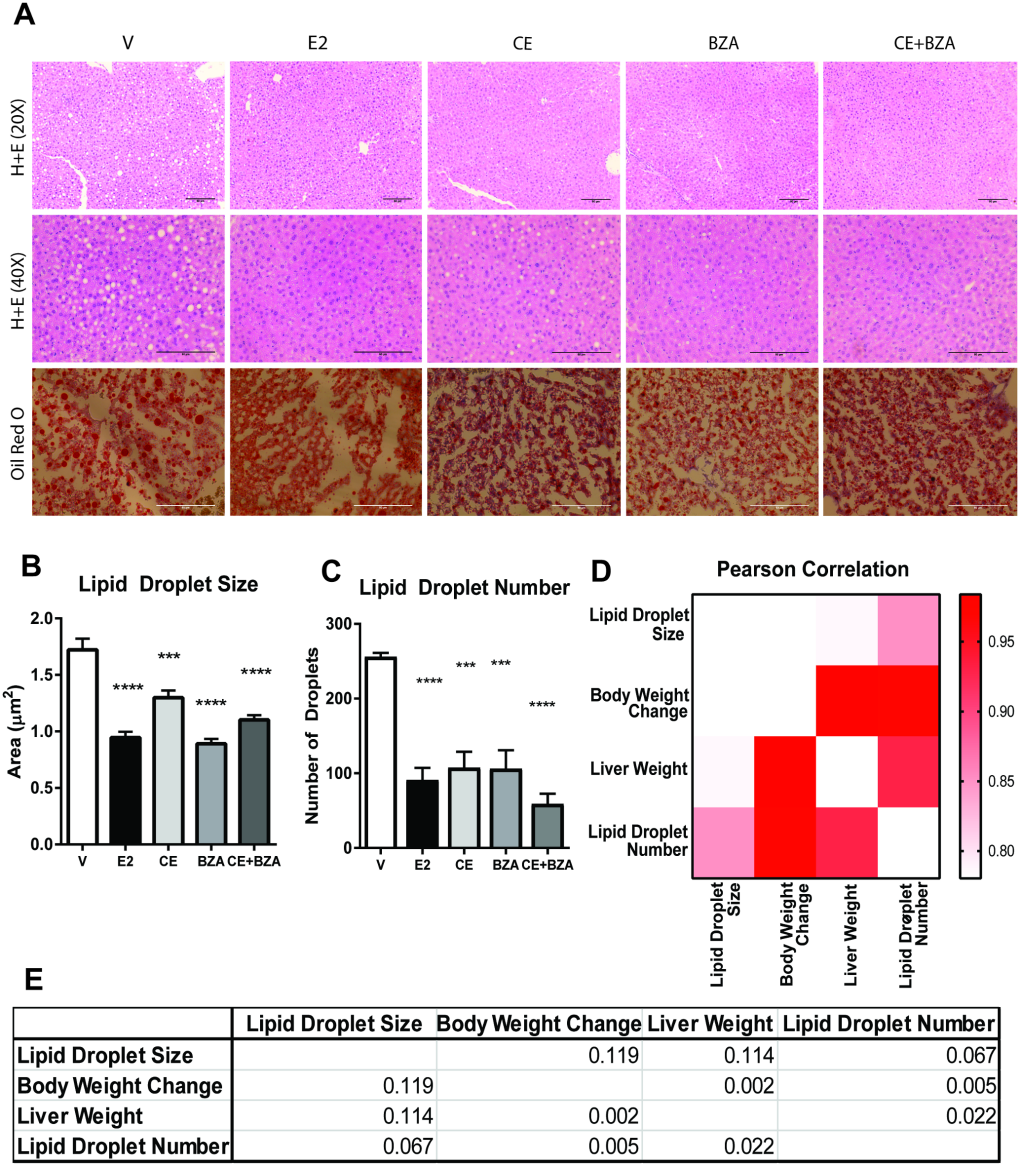
Lipid accumulation is decreased in mouse liver tissue after CE+BZA treatment. (A) H+E and Oil Red O staining of liver tissue from ovariectomized animals treated with various estrogens. Quantification of lipid droplet size (B) and number (C) by Fiji software. (D) Pearson correlations for lipid droplet size, body weight change, liver weight, and lipid droplet number were calculated. Statistical significance was established at α = 0.05. Pairwise t tests with a Bonferroni correction were used to identify treatments that were significantly different from Veh (***P <0.001, ****P<0.0001).

Since a statistically significant decrease in liver weight was prominent in the presence of estrogens, H&E and Oil Red O staining was performed on liver tissue to assess hepatic lipid accumulation **(**Fig 2A**)**. All treatments decreased the hepatic lipid accumulation and reduced the lipid droplet size **(**Fig 2A**)**. Quantification of the lipid droplet size and number revealed that CE+BZA was very effective in preventing steatosis in ovariectomized animals that were fed a high fat diet **(**Fig 2B and 2C**)**. We calculated Pearson correlation coefficients for lipid droplet size, body weight change, liver weight and lipid droplet number. This analysis showed that lipid droplet number was significantly correlated with the body weight change and liver weight. Additionally, lipid droplet number and lipid droplet size were slightly correlated (p-value of 0.067). Body weight and liver weight were also significantly correlated **(**Fig 2D and 2E**)**. These results validated that estrogen supplementation reduces liver weight by decreasing lipid accumulation.

### CE+BZA treatment regulates common and unique groups of genes in mice liver

**Fig 3:**
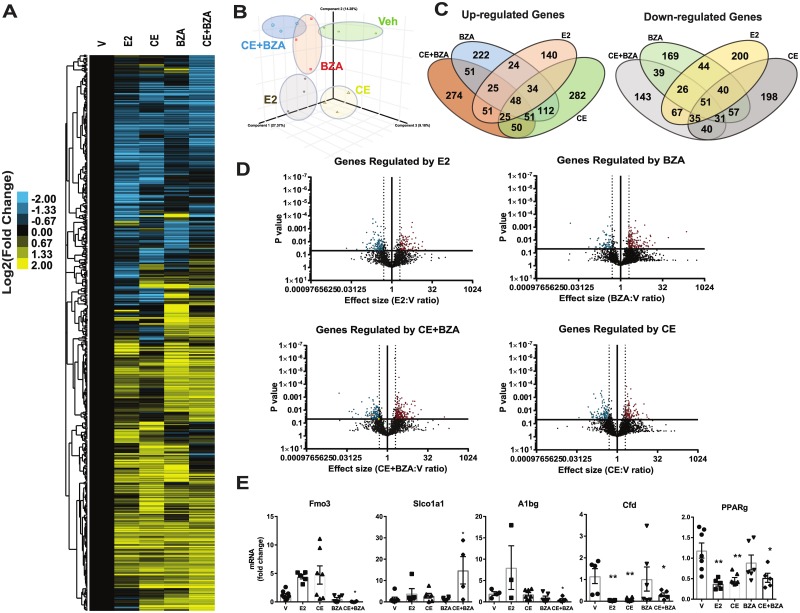
CE+BZA treatment regulates common and unique groups of genes in mice liver. Mice were treated with vehicle E2, CE only, BZA only, or CE+BZA for 6 weeks. (A) Heat map of relative fold change of genes from hepatic RNA-Seq analysis using genes that were significantly different then Veh group with P < 0.05 and expression fold change >2 (n = 3 biological replicates). Genes were clustered using Cluster 3 and visualized using Treeview Java (B) PCA analysis of differentially regulated genes from different treatment groups. (C) Venn diagram analysis of gene lists that are differentially regulated in various estrogen treatment groups compared to Veh group. (D) Volcano plots of gene expression data to show genes significantly affected by various estrogen treatments relative to Veh treatment. Statistical significance was determined at α = 0.05 and greater than a 2-fold change. Significantly downregulated genes are highlighted in blue and upregulated genes are highlighted in red. (E) qPCR validation of RNA-seq results. Statistical significance was established at α = 0.05. Results were analysed using unpaired t- tests (*P < 0.05, **P < 0.01).

Since we observed a significant impact of estrogen supplementation on liver weight and liver lipid deposition, next we assessed transcriptional changes induced by various estrogens in liver. RNA was extracted from mouse liver and RNA-seq was performed. Overall, treatments with various estrogens resulted in similar number of up- or down-regulated genes. CE+BZA up-regulates and down-regulates common as well as distinct groups of genes compared to E2, CE, and BZA **(**Fig 3A and 3D**)**. Hierarchical clustering **(**Fig 3A**)**, PCA analysis **(**Fig 3B**)** and Venn diagram analysis **(**Fig 3C**)** of differentially expressed genes in the presence of various estrogens compared to Veh treated animals showed that the CE+BZA treatment resulted in the most similar mode of gene regulation with BZA. Next, we validated several of the genes that we identified from this analysis, including Fmo3 (Flavin Containing Monooxygenase 3), Slco1a1 (solute carrier organic anion transporter family, member 1a1), A1bg (alpha-1-B glycoprotein), Cfd (Complement factor D) and Pparɣ (peroxisome proliferator activated receptor gamma) **(**Fig 3D**)**. Compared to individual treatments, CE or BZA, CE+BZA combination did not provide additional fold change suggesting that the effect of the combination is additive rather than synergistic.

**Fig 4:**
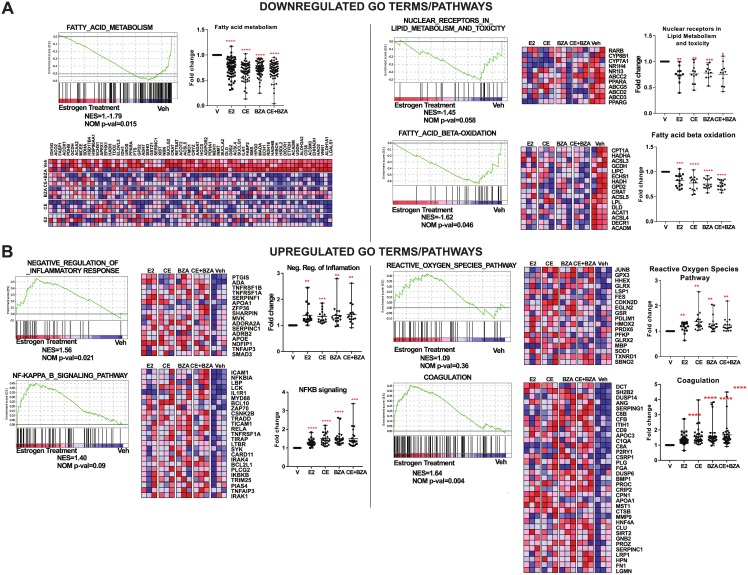
Gene ontology (GO) analysis of gene networks that are differentially regulated by CE+BZA treatment compared to Veh treatment. GSEA software was used to identify gene networks with specific functions. Gene set enrichment graphs for networks relevant to NAFLD biology that are (A) downregulated or (B) upregulated by various estrogen treatments are shown. Heatmap of expression of genes that contribute to enrichment score for each GO term are displayed. Fold change relative to Veh treatment of each gene was graphed (mean and range). All data were normally distributed. Statistical significance was established at α = 0.05. One-way Anova with a Dunn’s correction were used to identify treatments that were significantly different from Veh (*P < 0.05, **P < 0.01, ***P <0.001, ****P<0.0001).

Next, we used Cytoscape/ClueGO and GSEA analysis to identify gene networks that are associated with particular biological functions **(**Fig 4 and S2 Fig**)**. Consistent with the liver phenotype that we observed in Fig 2, all estrogen treatments resulted in a decrease in pathways related to fatty acid metabolism and lipid deposition **(**Fig 4A and S2 Fig**)**. Of note, there was also a decrease in fatty acid oxidation pathways **(**Fig 4A**, right lower panel)** suggesting that estrogen supplementation decreased the levels of available building blocks for lipid deposition without increasing the metabolism of the available lipids. In addition, our analysis identified an increase in pathways relevant to negative regulation of inflammatory response and reactive oxygen species pathways **(**Fig 4B**)**. The magnitude of regulation of genes related to these groups were higher in the CE+BZA group. Of note, in all the groups that received estrogen supplementation, genes associated with coagulation were increased **(**Fig 4B**, right lower panel)**. Overall, our analysis shows that estrogen supplementation counters the effects of ovariectomy and high-fat diets by reducing expression of lipid metabolism genes and increasing the expression of genes that reduce inflammation and reactive oxygen species.

### CE+BZA treatment distinctly alters blood metabolite composition

**Fig 5:**
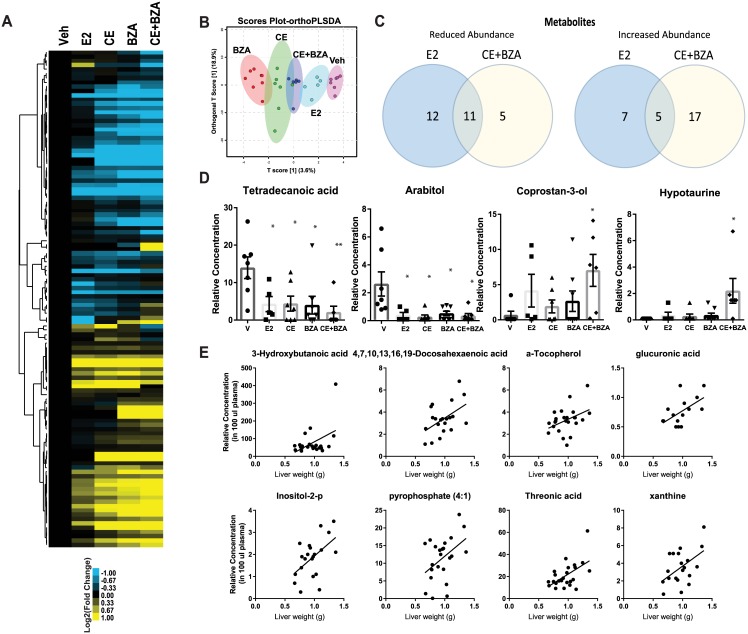
Whole metabolite analysis of plasma from animals that were treated with different ligands. Blood was harvested from mice six weeks after estrogen treatments. Plasma metabolites were identified by the GC-MS analysis. (A) Metabolomic data was clustered and visualized as a heatmap using Java TreeView. (B) ortho PLSDA analysis of the metabolite data using Metaboanalyst software. (C) Venn diagram analysis to identify distinct and common metabolites regulated by CE+BZA and E2 treatments. (D) Examples of metabolites that are regulated by various estrogen treatments. (E) Examples of metabolites that correlate with liver weight.

To understand changes in the composition of blood metabolites induced by various estrogens and validate the functional impact of hepatic gene expression changes, plasma from animals after 6 weeks of treatment with various estrogens were obtained and whole metabolomics analysis was performed **(**Fig 5A and S1 Table**)**. Whole metabolite profiling of plasma was able to segregate different treatment groups **(**Fig 5A and 5B**)**. Next, downregulated and upregulated metabolites were identified. Several of the metabolites were regulated by different estrogen treatments compared to Veh treatment **(**Fig 5C and 5D). To identify metabolites that are relevant to liver health, we correlated plasma metabolite levels with liver weight change. This analysis identified eight metabolites, several of which were shown to be associated with NAFLD, including ketone body 3-hydroxybutanoic acid [21](P = 0.03), 4,7,10,13,16,19-Docosahexaenoic acid[22] (P = 0.02), α-Tocopherol [23] (P = 0.04), threonic acid [24] (P = 0.005) and xanthine [25] (P = 0.02), which were all positively correlated with liver weight. **(**Fig 5E**)**. In summary, our data showed that estrogen supplementation after ovariectomy changes the abundance of metabolites related to NAFLD pathophysiology.

## Discussion

In the present study, we characterized the effects of estrogen supplementation after ovariectomy on hepatic gene expression and plasma metabolite profiles that are affected by high-fat diet. Gene networks and associated-plasma metabolites were consistent with the overall effects of estrogen treatments in terms of preventing low estrogen and high-fat diet-induced hepatic lipid accumulation.

Major factors that contribute to NAFLD progression are the accumulation of excessive hepatic fat and oxidative stress or inflammation [26–28]. These factors form the basis of the “2-hit hypothesis” and suggest that NAFLD is best treated by targeting both “hits”. Based on our analysis, several genes and metabolites associated with lipid metabolism pathways are downregulated by CE+BZA treatment, validating lower lipid deposition that was observed in the liver. We were able to validate several of the genes including Fmo3, Slco1a1, A1bg, Cfd and Pparɣ. Cfd has been shown the promote adipogenesis, and knockdown of Cfd resulted in inhibited lipid accumulation [29]. Pparɣ is a major regulator of lipogenesis and may also be stimulated by Cfd [29, 30]. In high-fat diet fed mice, Pparɣ expression is increased, leading to liver steatosis [31]. CE+BZA reverses this effect in our studies. Additionally, pathways associated with negative regulation of inflammation and protection against reactive oxygen species are improved with CE+BZA treatment. By modulating pathways associated lipid metabolism and inflammation, CE+BZA targets both “hits” of NAFLD pathogenesis and may effectively prevent symptoms leading to NAFLD and NASH without stimulating the uterus. We also observed that the coagulation related genes were upregulated in all estrogen treatment groups. This might explain the increased risk of thrombosis associated with oral HRT use [32–34]. Thus, our findings validate the beneficial metabolic effects of CE+BZA combination on liver health, yet might indicate a critical need for other delivery routes or novel estrogens that would provide similar profiles without increasing risk of thrombosis.

In our previous studies, we showed that some of the CE+BZA target genes were regulated by estrogen supplementation as early as 24h after treatment.[18, 19] In the current study, we monitored the hepatic transcriptomic changes after chronic estrogen supplementation. These changes are possibly a combination of gene circuits that are directly regulated by ER action, such as lipid metabolism, and other gene networks that are regulated in response to the dysregulated liver physiology, such as oxidative stress associated-pathways. Further studies are required to show the detailed kinetic properties of these gene regulation events. Yet, it is clear that the CE+BZA combination prevents dysregulation of these gene programs and their-associated pathological consequences.

Our metabolomics analysis identified various metabolites that are affected by various estrogen treatments. Several of these metabolites including ketone body 3-hydroxybutanoic acid [21], 4,7,10,13,16,19-Docosahexaenoic acid[22], α-Tocopherol [23], threonic acid [24] and xanthine [25] were previously shown to be associated with NAFLD. The metabolites tetradecanoic acid and coprostan-3-ol are also related to lipid and cholesterol biosynthetic pathways. Tetradecanoic acid/myristic acid is a fatty acid that is elevated in NAFLD [35]. Tetradecanoic acid is significantly decreased with CE+BZA treatment. Coprostan-3-ol is a cholesterol derivative poorly absorbed by the human intestine that is inversely associated with cholesterol levels in the liver [36]. Hypotaurine was significantly increased in CE+BZA treated animals. Hypotaurine supplementation has been shown to decrease oxidative stress and fibrosis [37]. Hypotaurine can also prevent steatohepatitis and hepatic lipid accumulation [37].

Overall, our datashows that estrogen supplementation improves ovariectomy and high-fat diet associated lipid accumulation in liver by decreasing expression of genes that regulate fatty acid metabolism and increasing expression of genes associated with inflammation and reversal of oxidative stress. Functional consequence of these changes are reflected in the plasma level of associated metabolites. When used together with the BZA, CE improves metabolic benefit while not stimulating the reproductive tissues and offer a relatively safer solution for postmenopausal symptoms and metabolic problems associated with estrogen deficiency. Our observations provide novel insights into the mechanism of NAFLD prevention by CE+BZA combination and suggest that further mechanistic and clinical studies are required validate these observations in postmenopausal women.

## Supporting information

S1 Table Relative abundance of metabolites from plasma of mice treated with various estrogens for six weeks. (XLSX)

S1 Fig Composition of conjugated estrogens (CE). (EPS)

S2 Fig Cytoscape networks of GO terms associated with CE+BZA up- or down-regulated genes. (A) Downregulated and (B) upregulated GO terms are shown in blue or red, respectively.
(EPS)
